# Recruitment Strategies in Registry-Based Observational Research: A Single-Center Experience in the United States from the HeartShare Study

**DOI:** 10.1016/j.yjcafi.2026.03.010

**Published:** 2026-05-13

**Authors:** VAISHNAVI KRISHNAN, RAMZI B. KIBBI, QUAN MAI, ELIZABETH MARQUEZ, LILI ZHAO, NICOLE CYRILLE-SUPERVILLE, GREGORY D. LEWIS, FARAZ S. AHMAD, NEELA THANGADA, SADIYA S. KHAN, LAURA J. RASMUSSEN-TORVIK

**Affiliations:** 1 Department of Medicine, Cardiology Division, Northwestern University Feinberg School of Medicine, Chicago, Illinois;; 2 Department of Preventive Medicine; Northwestern University, Chicago, Illinois;; 3 Sanger Heart and Vascular Institute, Atrium Health—Wake Forest, Charlotte, North Carolina; 4 Division of Cardiology, Massachusetts General Hospital, Boston, Massachusetts.

**Keywords:** Heart failure, study recruitment, health equity

## Abstract

**Background:**

Recruiting a representative sample of US adults is challenging, and strategies to improve recruitment have not been well evaluated. This study examined differences in consent rates across 3 recruitment approaches for the HeartShare registry at a single academic center.

**Methods:**

The HeartShare registry is a multicenter observational cohort enrolling adults age ≥30 years with or without heart failure. We included participants contacted at a single academic center between August 1, 2023 and August 1, 2024, comparing consent rates across 3 recruitment methods: (1) telephone; (2) electronic health record (EHR) messaging; or (3) in-person. Secondary analyses evaluated differences in consent rates within recruitment strategies by sociodemographic factors.

**Results:**

Among 9712 patients contacted (mean age, 72.0; standard deviation [SD] 13.1 years; 46.5% female), 4.2% consented. Of the 1439 patients contacted by telephone, 175 (12.1%) consented. Of the 8080 patients contacted through EHR messaging, 144 (1.8%) consented. Of the 193 contacted in-person, 79 (41%) consented. Compared with phone recruitment, EHR had the lowest consent rate (adjusted odds ratio [aOR], 0.13; 95% confidence interval [CI], 0.10–0.17) while in-person contact had the highest consent rate (aOR, 6.49; 95% CI, 4.42–9.53). In-person recruitment showed no significant differences in consent rates between Black and White participants (aOR, 0.69; 95% CI, 0.30–1.56). Among those approached via EHR or phone, Black individuals had significantly lower odds of consenting compared with White individuals (EHR: aOR, 0.31; 95% CI, 0.12–0.67; Phone: aOR, 0.36; 95% CI, 0.18–0.67).

**Conclusions:**

In-person recruitment achieved the highest consent rates across groups in this observational registry-based cohort. Although less time intensive, automated EHR recruitment was less effective in engaging underrepresented groups.

## Lay Summary

Researchers want to engage as many different types of people in clinical research so we can determine whether our research results apply to many different types of people. In this study, we looked at what percentage of people decided to enroll in our study depending on how they were approached to participate in the study. The highest percentage of people agreed to participate when we approached them in-person, but this method was time-consuming. Participants who self-identified as Black were less likely than other groups to agree to participate when approached using messages sent through the electronic health record or using the telephone.

## Introduction

Prioritizing the representativeness of study participants in health research is essential to ensure that findings are generalizable across populations of varying race, ethnicity, and socioeconomic status. Yet, recruitment in research studies often operates as a one-size-fits-all approach, which may result in a nonrepresentative sample among enrolled participants. This challenge has been increasingly recognized as a systemic problem, with many policies and initiatives attempting to address it.^1,2^ For example, the *New England Journal of Medicine* now requires authors to report on how well their study populations reflect those most affected by the disease, as representativeness is critical for both scientific validity and equitable care.^3^

In a retrospective analysis of over 200 heart failure (HF) observational studies and randomized controlled trials (RCTs), women comprised only 20% to 30% of participants despite representing roughly half of HF cases globally. Another systematic review found that only 15% of participants enrolled in RCTs for HF were non-White individuals.^4–6^ In HF with preserved ejection fraction (HFpEF), which is projected to become the dominant HF subtype in coming decades, women have nearly double the likelihood of developing HFpEF compared with HF with reduced ejection fraction (HFrEF;10.7% vs. 5.8%, respectively), and HFpEF disproportionately affects individuals with adverse social determinants of health.^7,8^ Moreover, additional tailoring of recruitment strategies may be helpful for patients with chronic diseases such as HF who are more likely to be older, have physical limitations, and a significant burden of comorbidities, which may impact enrollment and participation.^9,10^ Given the significant research gaps that remain in our understanding of HFpEF and its management, identifying the most effective recruitment methods is essential to optimize future recruitment efforts and ensure comprehensive representation in HF research studies.

Historically, telephone recruitment has been utilized and prioritized over in-person recruitment in many cases. However, recent factors such as the decline of landlines, along with a concurrent increase in robocalls and spam filters, have presented new challenges that have led to declining response rates to telephone contact, prompting researchers to explore other modalities to enhance participant engagement.^11^ Online recruitment, utilizing techniques such as email invitations and web advertisements, offers the benefits of being low cost and wide reaching while minimizing research staff time.^12^ However, this approach presents its own challenges, including its uncertain impact on the generalizability and validity of research findings. Participant skepticism towards online recruitment also remains a concern.^13^ The near-universal adoption of electronic health records (EHRs) with online messaging portals also offers a unique opportunity to leverage the ease and accessibility of electronic messaging to enhance recruitment.^14,15^

The multicenter HeartShare Study, funded by the National Heart, Lung, and Blood Institute (NHLBI), was recently launched to advance our understanding of the mechanisms underlying HFpEF.^16–18^ Recruitment for the online HeartShare registry (which includes patients with and without HFpEF) was conducted using a variety of methods, allowing us to evaluate different recruitment strategies for an online registry in an observational cohort study. Therefore, the primary objective of this analysis was to examine differences in response and consent rates for patients eligible for the registry contacted via telephone, EHR portal messages, or in-person at the Northwestern University HeartShare Clinical Center and to determine whether this differed across sociodemographic groups.

## Methods

### Study Sample

The HeartShare Study was established to advance the understanding of HFpEF through the identification of distinct HFpEF phenotypes, underlying disease mechanisms, and therapeutic targets based on phenotyping. The 2 research arms of HeartShare include the (1) online registry, which is the focus of the present analysis; and (2) in-person deep phenotyping visits consisting of noninvasive testing (including echocardiography, cardiopulmonary exercise testing, and imaging) and sample collections (blood, urine, stool, and saliva). Enrollment sites for HeartShare during the period of this analysis include Mass General Brigham (Boston, Massachusetts), Mayo Clinic (Rochester, Minnesota), Northwestern University (Chicago, Illinois), Atrium Health Wake Forest Medical Center (Winston-Salem, North Carolina), John Hopkins University (Baltimore, Maryland), University of California Davis (Davis, California), and the University of Pennsylvania (Philadelphia, Pennsylvania).^17,18^

Between August 1, 2023 and August 1, 2024, the Northwestern Clinical Center of the HeartShare Study employed various recruitment methods to consent patients in the registry, which consists of online questionnaires sent to participants each month. Any adults age 30 years or older are eligible to apply to participate in the HeartShare registry. Key exclusion criteria include a history of solid-organ transplantation, mechanical circulatory support, noncardiac cirrhosis, or an inability to provide written consent. Three recruitment methods were utilized: (1) telephone contact; (2) EHR portal messaging; and (3) in-person contact. All patient information and tracking of recruitment efforts were stored in REDCap, a secure data management system. Prior to their participation in Heart-Share, free and informed consent was obtained from all participants. This study was approved by the Northwestern University Institutional Review Board.

The first recruitment method was telephone recruitment, in which research coordinators called patients who were identified and prescreened using a local site-specific electronic data warehouse: the Northwestern Medicine Enterprise Data Warehouse (NMEDW), a comprehensive integrated repository of clinical and research data sources from the Northwestern University and Northwestern Medicine hospital networks. The NMEDW provided a report with contact information for individuals who had (1) at least 1 visit in the Cardiology clinic within the past 30 days based on a selected list of clinicians who primarily care for patients with HFpEF and (2) had a diagnosis of HF based on International Classification of Diseases (ICD) codes (ICD-9: 398.91, 402.x1, 404.x1, 404.x3, and 428.xx; ICD-10: I09.81, I11.0, I13.0, I13.2, and I50.xx). Coordinators then contacted the patients via telephone, explained the study details using a standardized script, and invited them to participate. If the patient agreed, the coordinator sent an email with a link to sign up for the study on the Eureka Research Platform, a digital tool used across all the sites in the HeartShare Study and designed to enhance health research by enabling remote recruitment and mobile data collection.^19^ On Eureka, the patient could create an account and sign the consent form online to officially participate in the study. Participants recruited by telephone were also, in some limited cases, identified initially through various methods such as physician referrals, clinic visits, self- or word-of-mouth referrals, family members, and other channels.

The second method of recruitment was EHR portal messaging to patients who met eligibility criteria for HeartShare and received care from clinicians who were not on the telephone recruitment list (to avoid duplication). Approximately 400 secure messages were sent weekly to patients through the EHR patient portal, inviting them to participate in the HeartShare Study. The study invitations were sent directly to the patients as authorized messages within the secure portal. This message was sent only once and provided a brief study overview alongside an option for recipients to express interest in participation. For those who expressed interest, coordinators sent a follow-up email with more study details and a request for confirmation of interest. The participants who confirmed their interest via email were sent an invite link to Eureka, where they had the option of creating an account and signing the consent form to officially participate in the study. To diversify recruitment, patient portal messaging was additionally directed based on demographic information in the health record. From December to May 2024, 70% of the weekly EHR messages for recruitment were selectively directed toward non-White populations. In May 2024, EHR message targeting of non-White populations was reduced to 50%, and in June 2024 all directed recruitment based on race and ethnicity was discontinued, with ongoing messaging based on random selection of eligible participants.

The third recruitment method was in-person recruitment, which involved research coordinators using electronic tablets to consent patients in inpatient floor settings, waiting rooms, or outpatient clinics. Coordinators approached patients, introduced the study, and offered the option to consent on-site or via an email invitation to consent on the Eureka website. After the consent was signed, an automated follow-up email was sent for survey access. In waiting rooms, coordinators approached patients post check-in, introduced the study, and proceeded with the consent process if the patient was eligible and interested. For inpatient floors and outpatient clinics, patients were prescreened to assess eligibility for the study before being approached by coordinators.

### Covariates

Data on age, race and ethnicity, and 9-digit ZIP code from residential address were extracted directly from the EHR for all participants. Race and ethnicity were generally self-reported by patients. Gender was defined as the socially constructed designation documented in the electronic health record (male or female), which may not reflect sex assigned at birth. Based on ZIP code, the area deprivation index (ADI) was calculated for all participants and examined as quintiles (1st quintile = least disadvantaged, 5th quintile = most disadvantaged).^20,21^

### Statistical Analysis

Chi-square or Fisher’s exact tests were performed, as appropriate given sample sizes, to assess the significance of associations between recruitment method and response rates. Analysis of variance (ANOVA) tests were conducted for the continuous variable age to assess differences in mean age across recruitment methods. Logistic regression models were used to compare the odds of consent among participants across recruitment methods. Both unadjusted and adjusted models were examined to assess the associations between demographic characteristics, recruitment methods, and consent. To assess effect modification, interaction terms were included for recruitment method and covariates to assess whether the association between recruitment method and consent varied by demographic factors. Separate logistic regression analyses were conducted stratified by recruitment method. Statistical analyses were conducted using R Statistical Software version 4.2.4 (R Foundation for Statistical Computing, Vienna, Austria).

## Results

Baseline demographics for participants contacted for participation in the HeartShare Registry Study at the Northwestern Clinical Center between August 1, 2023 and August 1, 2024 are summarized in Table 1. Among the 9712 total participants contacted through 1 of the 3 recruitment methods, 1439 participants were contacted via telephone, 8080 via EHR portal messaging, and 193 in-person. Across all recruitment methods, the majority of patients contacted identified as male, non-Hispanic, and White. Among the 403 participants who consented, 175 were initially contacted via telephone, 149 via EHR, and 79 in-person, with significant differences in consent by recruitment method for age, race, and ethnicity (Table 2).

**Fig. 1.**
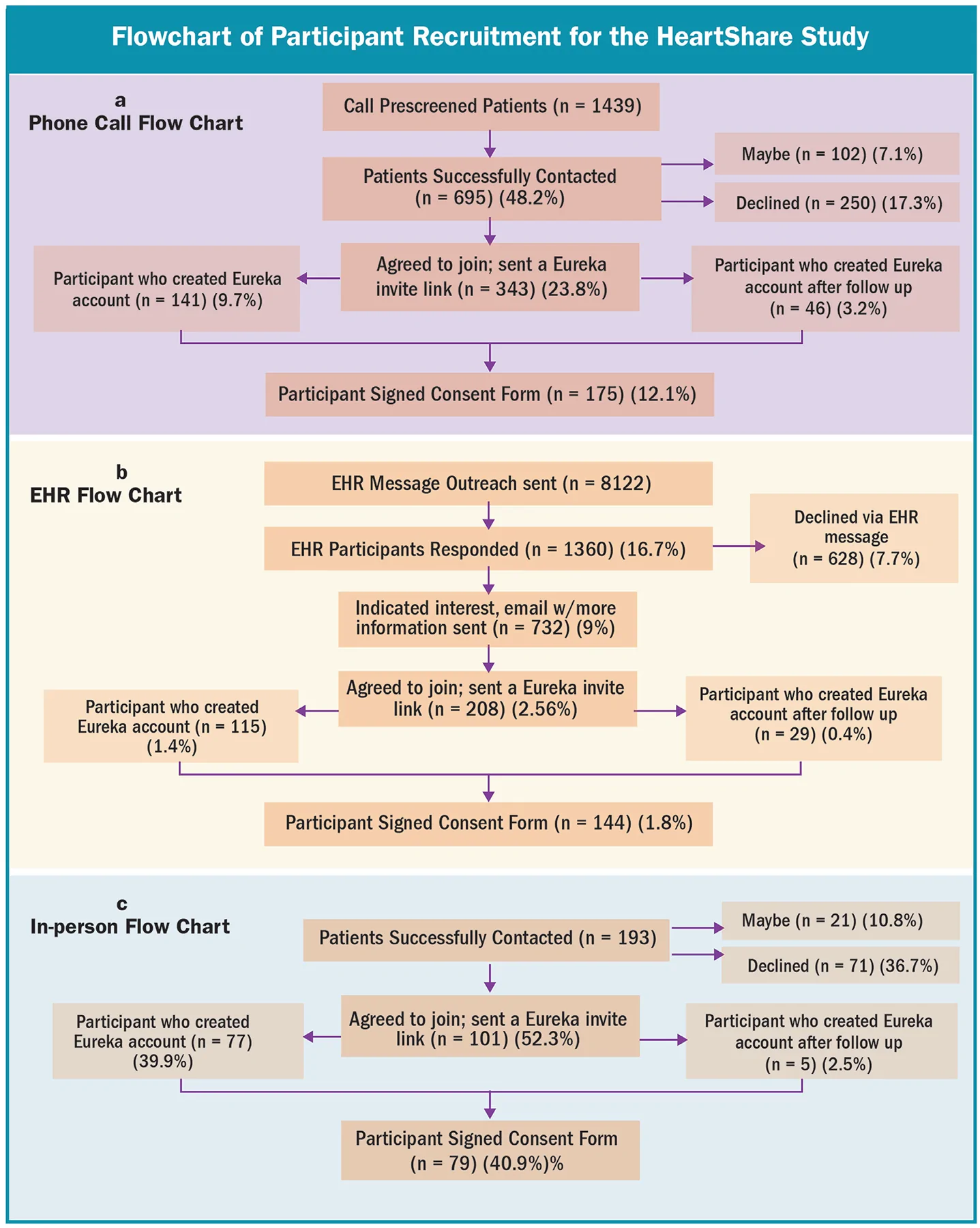
Flowcharts demonstrating the recruitment process for the HeartShare Study, including the number of individuals contacted through each method (phone, electronic health record, and in-person), the number who accepted participation, and the final number who consented. Each recruitment pathway is illustrated separately to highlight differential yields across methods.

Fig. 1 presents recruitment flowcharts for telephone, EHR messaging, and in-person recruitment, and displays each stage of the recruitment process, including screening, outreach, contact, agreement for participation, and consent. There was variation in the number of participants contacted with each method, which reflects the inherent nature and resource requirements of each recruitment method. For phone recruitment (Fig. 1A), 48.2% (n = 695) of the initial prescreened population (n = 1439) were successfully contacted. Of those successfully contacted (defined as a participant who either answered the phone or returned a call), 343 people agreed to participate and were sent an invite link to sign up for the study. Ultimately, 175 participants (12.1% of the initial sample) completed the consent to join the HeartShare registry through phone recruitment. For EHR recruitment (Fig. 1B), 8122 participants were contacted and 16.7% (n = 1360) responded, with 208 agreeing to join and 144 (1.8% of the initial sample) completing the consent. For in-person recruitment (Fig. 1C), 193 participants were contacted and 79 (40.9% of the initial sample) consented to the study.

Response, defined as a participant ultimately completing the consent to join the study, varied significantly across different recruitment methods and demographic groups in the HeartShare Study. In a multivariable regression model examining predictors of consent, a significant interaction between race and ethnicity and recruitment method was observed (*P* = .04). When analyses were stratified by recruitment method, individuals identifying as Black were significantly less likely than those identifying as white to consent via phone (adjusted odds ratio [aOR], 0.36; 95% CI, 0.18–0.67; Appendix Table 1), and EHR (aOR, 0.31; 95% CI, 0.12–0.67; Appendix Table 2) recruitment methods. In contrast, no significant difference in odds of consent was observed between individuals identifying as Black or White when recruitment occurred in-person (aOR, 0.69; 95% CI, 0.30–1.56; Appendix Table 3). For phone recruitment, individuals identifying as Asian (aOR, 0.20; 95% CI, 0.03–0.66) or male (aOR, 0.66; 95% CI, 0.46–0.95) were also less likely to consent compared with individuals identifying as White or female, respectively. Flowcharts depicting individuals contacted, responded, and ultimately consented stratified by race are presented in Appendix Figs. 1A and 1B; they demonstrate lower overall consent rates among individuals identifying as Black compared with White.

There was a significant interaction between ADI and recruitment method in the multivariable regression model examining predictors of consent (*P* = .002). Participants who resided in the most disadvantaged areas were significantly less likely to consent via EHR outreach compared with least disadvantaged areas (Quintile 5 vs. 1: aOR, 0.31; 95% CI, 0.14–0.60; Appendix Table 2). In contrast, telephone and in-person recruitment demonstrated no significant differences in participation rates according to residing in a more or less disadvantaged area (Appendix Tables 1 and 3).

## Discussion

In this prospective evaluation of multimodal recruitment strategies in the online HeartShare registry, there was significant variability in response and consent rates when contacting eligible patients by telephone, EHR portal messages, and in-person. Moreover, there were significant sociodemographic differences when using lower-touch recruitment methods via telephone and portal messages compared with in-person recruitment. These findings highlight the importance of prioritizing in-person recruitment even when considering multiple recruitment strategies to ensure inclusivity in observational registry-based research.

Each recruitment method required varying levels of effort and yielded different rates of patient consent into the online registry. This study found that in-person recruitment, although being the most time-intensive, resulted in the highest consent rates across all sociodemographic groups, highlighting its potential to engage diverse populations. One study from 2017 examining recruitment at 4 urban health care centers showed that in-person recruitment resulted in higher response rates when compared with letters and emails, particularly with minority populations.^22^ This method offers key advantages, such as fostering cultural sensitivity and building trust with communities that may harbor skepticism toward research due to historical or systemic inequities. However, few studies have systematically reported or examined consent rates across different recruitment strategies. This has been highlighted by federal and national funding agencies as well as publishing groups as a priority to understand and improve representativeness in observational studies and clinical trials. Culturally competent and diverse research leadership also plays a critical role in advancing these efforts. Engaging diverse investigators, patients, and community members in study design, communication, and consent processes helps research teams address socioeconomic and cultural barriers, reduce bias, and pose research questions that reflect the needs of underrepresented populations. This allows a shift in research from simple enrollment to authentic partnership throughout all stages of the study.^23^

Remote or lower-touch methods, such as email, often yield lower response rates among minority populations, potentially due to access barriers or mistrust. In our recruitment efforts, EHR messaging demonstrated the lowest response rate among all 3 recruitment methods, higher drop-off rates from contacted to consented, and reduced success in recruiting patients from diverse racial and ethnic backgrounds. A 2024 study evaluating the use of EHR-based recruitment through the MyChart patient portal across multiple specialties (including psychiatry, neurology, and public health sciences) at the University of Rochester Medical Center reported an overall response rate of 23%, with one-third of respondents expressing interest in learning more. Notably, response rates among Black and Hispanic participants were approximately half those observed in White participants, highlighting disparities in engagement with EHR messaging for research recruitment.^24^ Additionally, several studies have noted significant differences in EHR portal activation and usage across sociodemographic characteristics—including age, sex, race and ethnicity, primary language, insurance status, educational attainment, and access to technology—which results in a sample not representative of the source population when recruiting using EHR messaging.^25–27^

It is important to note that despite low absolute response rates of consent, automated EHR messaging enabled our study to efficiently contact thousands of participants, thus yielding a similar magnitude of consented participants compared with phone recruitment overall. However, the findings of this study caution against the rapid and exclusive adoption of this approach in research. More work is needed to understand how representativeness can be improved among the smaller sample of participants who do respond via EHR. In addition, EHR recruitment may disadvantage patients with non-English language preference (NELP).^28^ Patients with a NELP face systemic barriers that limit their access to clinical studies, including inadequate interpreter services, longer encounter times, and lack of culturally and linguistically concordant staff. These challenges contribute to lower participation rates and are rarely accounted for in trial reporting or national registry data. This underscores the importance of prioritizing primary language concordance during recruitment.^29^

In contrast, in-person recruitment, while more time-intensive, achieved markedly higher consent rates overall and across all demographic groups in our recruitment efforts. These findings highlight the importance and need for time, personnel, and resources to ensure equitable recruitment. This also emphasizes the need for thoughtfully designed strategies to enhance engagement among underrepresented populations, particularly when using phone and EHR-based approaches. A more effective model, combining technological efficiency with personalized, community-engaged outreach, could better support inclusive enrollment and generate valid generalizable data.^30^ Moreover, this work underscores the need for standardized reporting of recruitment strategies in addition to evaluation of different approaches in systematic prospective studies.

### Limitations

This study has several limitations. First, the single-center design at an academic medical center may limit its generalizability to other nontertiary or rural settings. Second, there is a potential for misclassification of race and ethnicity, which may have been assigned by a health worker or may be missing in the EHR. Third, the effort across recruitment strategies, including the time spent for each method, was not tracked prospectively or assigned randomly, which limits inferences about the enrollment strategies. Fourth, targeted outreach efforts were implemented to increase the representativeness of the patient population in the registry, which may bias the findings toward greater rates of underrepresented groups consenting.

## Conclusion

Our analysis of recruitment for the online HeartShare registry demonstrated that multimodal recruitment strategies require prospective evaluation to effectively engage diverse populations and ensure inclusivity in registry-based observational research. While each method has unique strengths and limitations, integrating strategies such as phone calls, EHR portal messaging, and in-person recruitment may ultimately improve diversity in clinical research studies in the United States. Future research should continue to focus on developing tailored approaches to enhance engagement in partnership with groups underrepresented in research.

## Supplementary Material

Supplementary Material

Supplementary material associated with this article can be found in the online version at doi:10.1016/j.yjcafi.2026.03.010.

## Figures and Tables

**Table 1:** Sociodemographic characteristics of patients contacted to participate in the HeartShare Study across all recruitment methods.

	Telephone Recruitment	Electronic Health Record Recruitment	In-Person Recruitment	*P* value
**Number of participants**	1439	8080	193	
**Age, y, mean (standard deviation)**	71.6 (12.8)	72.2 (13.1)	66.9 (12.1)	<**.001**
**Gender (%)**				
Male	751 (52.1)	4325 (53.5)	102 (52.8)	.98
Female	657 (45.7)	3755 (46.5)	87 (45.1)	
Not available	31 (2.2)	0 (0)	4 (2.1)	
**Race (%)**				
Native American/Alaska Native	4 (0.3)	15 (0.2)	0 (0)	<**.001**
Asian	63 (4.4)	320 (4)	4 (2.1)	
Black	258 (17.9)	1235 (15.3)	60 (31.1)	
Native Hawaiian/Other Pacific Islander	0 (0)	10 (0.1)	1 (0.5)	
White	948 (65.9)	5735 (71)	101 (52.3)	
Unknown or not reported	166 (11.5)	761 (9.4)	27 (14.0)	
**Ethnicity (%)**				
Not Hispanic or Latino	1262 (87.6)	7137 (88.3)	158 (81.9)	**.001**
Hispanic or Latino	87 (6.0)	546 (6.8)	19 (9.8)	
Unknown or not reported	90 (6.4)	397 (4.9)	16 (8.3)	

*P* values compare each sociodemographic characteristic among patients contacted for the HeartShare Study across recruitment methods. Statistical significance was determined using a Chi-squared or Fisher’s exact test, as appropriate, for categorical variables, and using an analysis of variance for continuous variables. Boldface indicates statistical significance (*P* < .05).

**Table 2:** Sociodemographic characteristics of consented patients in the HeartShare Study across all recruitment methods.

	Telephone Recruitment	Electronic Health Record Recruitment	In-Person Recruitment	*P* value
**Number of participants**	175	149	79	
**Age, y, mean (standard deviation)**	71.6 (12.1)	69.3 (11.7)	66.1 (12.7)	<**.001**
**Gender (%)**				
Male	83 (47.4)	88 (59.1)	42 (53.2)	.98
Female	92 (52.6)	61 (40.9)	37 (46.8)	
Not available				
**Race (%)**				
Native American/Alaska Native	1 (0.6)	0 (0)	0 (0)	<**.001**
Asian	2 (1.2)	2 (1.3)	0 (0)	
Black	17 (9.7)	6 (4.0)	23 (29.1)	
Native Hawaiian/Other Pacific Islander	0 (0)	1 (0.7)	0 (0)	
White	149 (85.1)	136 (91.3)	45 (57)	
Unknown or not reported	6 (3.4)	4 (2.7)	11 (13.9)	
**Ethnicity (%)**				
Not Hispanic or Latino	163 (93.1)	147 (98.6)	66 (83.6)	**.0017**
Hispanic or Latino	4 (2.3)	1 (0.7)	8 (10.1)	
Unknown or not reported	8 (4.6)	1 (0.7)	5 (6.3)	

*P* values compare each sociodemographic characteristic among consented participants in the HeartShare Study across recruitment methods. Statistical significance was determined using a Chi-squared or Fisher’s exact test, as appropriate, for categorical variables, and with analysis of variance for continuous variables. Boldface indicates statistical significance (*P* < .05).
