# Supplementary material for: Recruitment Strategies in Registry-Based Observational Research: A Single-Center Experience in the United States from the HeartShare Study
Source: J Card Fail Intersect. Author manuscript; Available in PMC 2026 Sep 16. (PMC13576520; doi:10.1016/j.yjcafi.2026.03.010)
Supplement: Supplementary file 1 [file NIHMS2192393-supplement-Supplementary_Material.docx]

**SUPPLEMENTAL MATERIAL**

Krishnan V*, Kibbi RB*, Mai Q, et al. Recruitment strategies in registry-based observational research: A single center experience in the United States from the HeartShare Study.

**These authors contributed equally*

**SUPPLEMENTAL TABLES**

**Appendix Table 1: Associations of sociodemographic characteristics and recruitment methods with consent rates among patients approached via telephone recruitment.** Odds ratios (OR) and 95% confidence intervals (CI) were calculated from unadjusted or adjusted logistic regression models that included age (continuous), gender, race, ethnicity, and ADI quintile as covariates.

|  | **Unadjusted OR (95% CI)** | **P-Value** | **Adjusted OR (95% CI)** | **P-Value** |
| --- | --- | --- | --- | --- |
| **Age (per 1-year increase)** | 1.00 (0.99,1.01) | p = 0.352 | 0.99 (0.97, 1.00) | p = 0.350 |
| **Gender** |  |  |  |  |
| Female | Reference | **p = 0.022** | Reference | **p= 0.027** |
| Male | 0.76 (0.56, 1.05) |  | 0.66 (0.46, 0.95) |  |
| **Race** |  |  |  |  |
| White | Reference | **p < 0.001** | Reference | **p < 0.001** |
| Asian | 0.18 (0.03, 0.57) |  | 0.20 (0.03, 0.66) |  |
| Black | 0.38 (0.22, 0.62) |  | 0.36 (0.18, 0.67) |  |
| Unknown or Not Reported | 0.25 (0.10, 0.53) |  | 0.29 (0.09, 0.79) |  |
| **Ethnicity** |  |  |  |  |
| Not Hispanic or Latino | Reference | p = 0.639 | Reference | p = 0.6551 |
| Hispanic or Latino | 0.32 (0.10, 0.79) |  | 0.64 (0.17, 1.80) |  |
| Unknown or Not Reported | 0.94 (0.39, 1.99) |  | 1.14 (0.31, 3.34) |  |
| **ADI** |  |  |  |  |
| ADI quintile 1 | Reference | p = 0.543 | Reference | p = 0.179 |
| ADI quintile 2 | 0.91 (0.61, 1.36) |  | 0.98 (0.60, 1.60) |  |
| ADI quintile 3 | 0.55 (0.32, 0.93) |  | 0.93 (0.56, 1.51) |  |
| ADI quintile 4 | 0.45 (0.19, 0.94) |  | 0.671 (0.35, 1.20) |  |
| ADI quintile 5 | 0.57 (0.17, 1.50) |  | 0.721 (0.37, 1.34) |  |

**Appendix Table 2:** **Associations of sociodemographic characteristics and recruitment methods with consent rates among patients approached via EHR recruitment.** Odds ratios (OR) and 95% confidence intervals (CI) were calculated from unadjusted or adjusted logistic regression models that included age (continuous), gender, race, ethnicity, and ADI quintile as covariates.

|  | **Unadjusted OR (95% CI)** | **P-Value** | **Adjusted OR (95% CI)** | **P-Value** |
| --- | --- | --- | --- | --- |
| **Age (per 1-year increase)** | 0.98 (0.97, 0.99) | **p < 0.001** | 0.97 (0.96, 0.98) | **p < 0.001** |
| **Gender** |  |  |  |  |
| Female | Reference | p = 0.957 | Reference | p = 0.9732 |
| Male | 1.26 (0.91, 1.75) |  | 1.00 (0.70, 1.42) |  |
| **Race** |  |  |  |  |
| White | Reference | **p < 0.001** | Reference | **p= 0.007** |
| Asian | 0.40 (0.10, 1.05) |  | 0.40 (0.09, 1.08) |  |
| Black | 0.20 (0.08, 0.42) |  | 0.31 (0.12, 0.67) |  |
| Unknown or Not Reported | 0.16 (0.03, 0.50) |  | 0.24 (0.01, 1.08) |  |
| **Ethnicity** |  |  |  |  |
| Not Hispanic or Latino | Reference | **p < 0.001** | Reference | **p < 0.001** |
| Unknown | 0.28 (0.05, 0.89) |  | 0.20 (0.02, 0.87) |  |
| Hispanic or Latino | 0.09 (0.01, 0.40) |  | 0.0000002 (0, 0.63) |  |
| **ADI** |  |  |  |  |
| ADI quintile 1 | Reference | **p < 0.001** | Reference | **p < 0.001** |
| ADI quintile 2 | 0.58 (0.39, 0.87) |  | 0.78 (0.49, 1.23) |  |
| ADI quintile 3 | 0.44 (0.26, 0.70) |  | 0.52 (0.30, 0.86) |  |
| ADI quintile 4 | 0.30 (0.12, 0.62) |  | 0.58 (0.33, 0.96) |  |
| ADI quintile 5 | 0.12 (0.007, 0.56) |  | 0.31 (0.14, 0.60) |  |

**Appendix Table 3:** **Associations of sociodemographic characteristics and recruitment methods with consent rates among patients approached via in-person recruitment.** Odds ratios (OR) and 95% confidence intervals (CI) were calculated from unadjusted or adjusted logistic regression models that included age (continuous), gender, race, ethnicity, and ADI quintile as covariates.

|  | **Unadjusted OR (95% CI)** | **P-Value** | **Adjusted OR (95% CI)** | **P-Value** |
| --- | --- | --- | --- | --- |
| **Age (per 1-year increase)** | 0.99 (0.97, 1.01) | p = 0.482 | 0.98 (0.95, 1.01) | p = 0.294 |
| **Gender** |  |  |  |  |
| Female | Reference | p = 0.85 | Reference | p = 0.88 |
| Male | 0.95 (0.53, 1.69) |  | 1.04 (0.53, 2.06) |  |
| **Race** |  |  |  |  |
| White | Reference | p = 0.989 | Reference | p = 0.15 |
| Black | 0.77 (0.40, 1.48) |  | 0.69 (0.30, 1.56) |  |
| Unknown or Not Reported | 1.04 (0.40, 2.62) |  | 1.13 (0.24, 5.19) |  |
| **Ethnicity** |  |  |  |  |
| Not Hispanic or Latino | Reference | p = 0.987 | Reference | p = 0.97 |
| Hispanic or Latino | 1.01 (0.37, 2.64) |  | 1.14 (0.25, 4.90) |  |
| Unknown | 0.80 (0.20, 2.75) |  | 1.30 (0.18, 9.74) |  |
| **ADI** |  |  |  |  |
| ADI quintile 1 | Reference | p = 0.695 | Reference | p = 0.53 |
| ADI quintile 2 | 0.84 (0.35, 2.01) |  | 0.44 (0.12, 1.45) |  |
| ADI quintile 3 | 1.21 (0.51, 2.91) |  | 0.88 (0.31, 2.45) |  |
| ADI quintile 4 | 1.18 (0.38, 3.60) |  | 1.53 (0.55, 4.31) |  |
| ADI quintile 5 | 0.82 (0.22, 2.80) |  | 0.90 (0.33, 2.43) |  |

**SUPPLEMENTAL FIGURES**

**Appendix Figure 1: Flowchart of EHR-Based Recruitment Stratified by Race.**
Flowcharts demonstrate the flow of participants identified and contacted through the Electronic Health Record (EHR) among White (a) and Black (b) participants, including the number of individuals contacted, responding, and ultimately consented.
